# *De novo* mutations discovered in 8 Mexican American families through whole genome sequencing

**DOI:** 10.1186/1753-6561-8-S1-S24

**Published:** 2014-06-17

**Authors:** Heming Wang, Xiaofeng Zhu

**Affiliations:** 1 Department of Epidemiology and Biostatistics, Case Western Reserve University, 10900 Euclid Ave, Cleveland, OH 44106-4945, USA

## Abstract

*De novo* mutations enrich the sequence diversity and carry the clue of evolutional selection. Recent studies suggest the *de novo* mutations could be one of the risk factors for complex diseases. We conducted a survey of *de novo* mutations using the whole genome sequence data but only available on the odd autosomes of Mexican American families provided by Genetic Analysis Workshop 18. We extracted 8 three-generation families who have sequencing data available from 20 large pedigrees. By comparing the known single nucleotide variants (SNVs) in dbSNP129 and the *de novo* variants transmitted in the Mexican American families, we were able to estimate a *de novo* mutation rate of 1.64(±0.42) × 10^−8 ^per position per haploid genome. This result is consistent with the estimates in literature that required many extensive validation efforts, such as genotyping and further resequencing. Our analysis suggests the importance of using family samples for studying rare variants.

## Background

*De novo *mutations enrich the sequence diversity and carry the clue of evolutional selection [1]. Because of the technological advances in whole genome sequencing, genome-wide *de novo *mutation survey becomes possible. Recent studies show that *de novo *mutations, including *de novo *copy number variations, are strongly associated with multiple diseases, such as autism and schizophrenia [2]. Currently *de novo *mutations are often studied in family trios by comparing the parents' and child's whole genome sequence data, as well as the publicly available dbSNP database [3]. Variants observed in offspring, but not in their parents, are often considered as potential *de novo *mutations. However, even highly accurate sequencing data will have inevitable errors that lead to false variant callings and possible mendelian errors. Therefore, the *de novo *mutation candidates observed by comparing offspring's and their parents' sequencing data can be false positive [4]. Thus, researchers often resequence or genotype the candidates to confirm the true *de novo *mutations [1-4]. This procedure could be time and money consuming. Here we propose an approach using 3-generation families to detect *de novo *mutations (a) using the parents and grandparents to search for *de novo *mutation candidates, and (b) using offspring sequence data to confirm true *de novo *mutations. We applied this approach to the Genetic Analysis Workshop 18 (GAW18) data and found our results consistent with previous genotyping and further resequencing validation efforts. This result suggested our approach is reliable. With the continuously decreasing cost of whole genome sequencing, this approach should be efficient to detect *de novo *mutations.

## Methods

**Figure 1:**
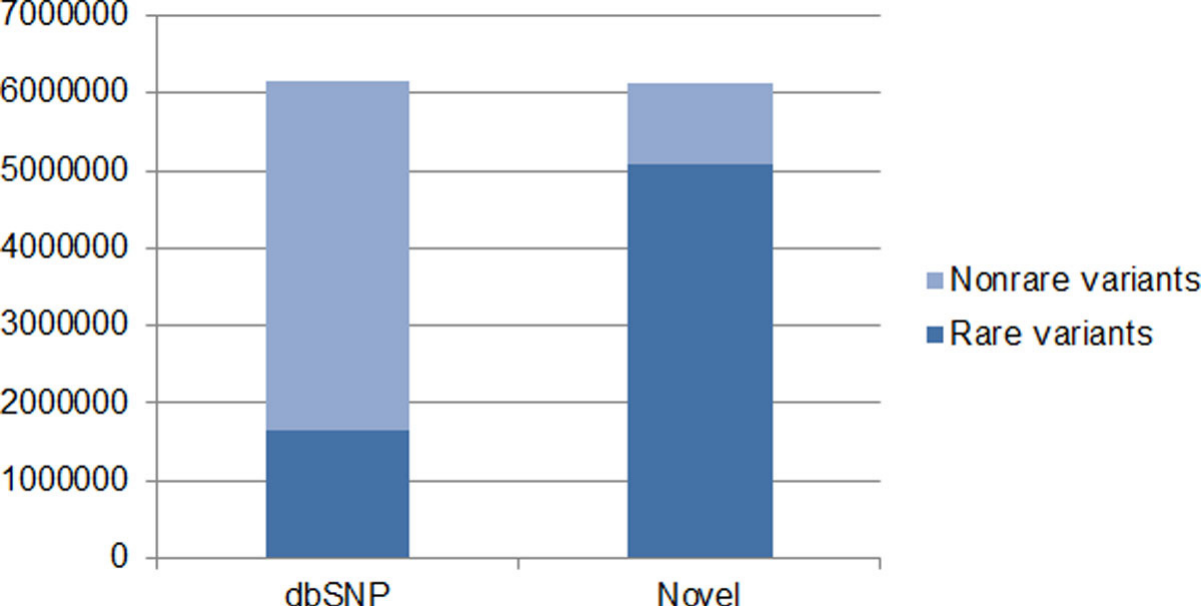
**Comparison between the distribution of SNVs in dbSNP129 and novel SNVs**.

**Figure 2:**
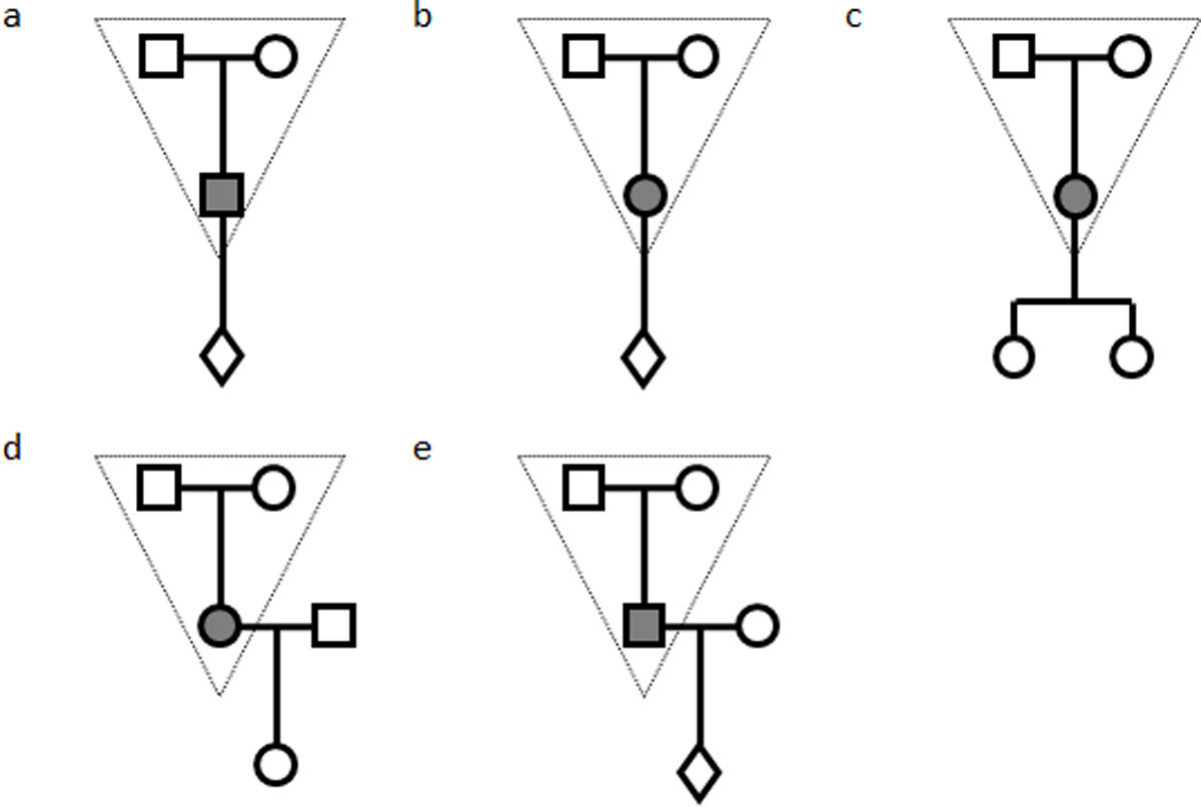
**A summary of selected family types**. We identified 1 a family, 2 b families, 1 c family, 1 d family, and 3 e families. The upper trios (in the dashed triangles) are used to identify *de novo *mutation candidates, and the third generations are used to confirm a true *de novo *mutation. Family a and e measure the *de novo *mutations in males. Family b, c, and d measure the *de novo *mutations in females.

GAW18 data include 20 large Mexican American pedigrees as part of the Type 2 Diabetes Genetic Exploration by Next-generation sequencing in Ethnic Samples (T2D-GENES) project. Whole genome sequence data on the odd autosomes are provided to the GAW18 participants. Our analysis focused on the 464 individuals who were whole genome sequenced, resulting in 12 million SNVs. Among those, more than 6.1 million SNVs are novel and not present in dbSNP129. Among the novel SNVs, 5,086,136 SNVs have minor allele frequencies less than 0.5% (Figure 1). As our goal is to detect *de novo *mutations, our analysis is restricted to these novel and rare SNVs in order to reduce the false-positive rate. When a real *de novo *mutation is observed in an individual, there is a 50% probability of it being transmitted to each of the individual's children. Thus, the transmission of variants from an individual to the individual's offspring can be used as a validation procedure in detecting the *de novo *mutations. Therefore, we selected families with sequenced data available for at least 3 generations. A total of 8 three-generation families were selected (Figure 2). For each of the families in Figure 2, we examined every rare and novel variant and considered it as a *de novo *mutation candidate if it is present in a parent (the child in the triangle) but absent in both grandparents. We next examined whether a *de novo *mutation candidate is transmitted from a parent to the parent's offspring. Only a *de novo *mutation candidate who transmitted to his/her offspring is declared as a true *de novo *mutation. Among the 8 families in Figure 2, 4 families (including 1 **a **family and 3 **e **families) were used to identify *de novo *mutations in males, and 4 families (including 2 **b **families, 1 **c**, and 1 **d **families) were used to identify *de novo *mutations in females, depending on whether the parent is male or female. We further categorized the 8 families into 2 family types according to the number of offspring: type I included families **a**, **b**, **d**, and **e**, and type II included family **c**. Let *N_o _*be the number of *de novo *mutations observed in a family and *L *be the sequence length of all odd autosomes in human. For a type I family, the total number of *de novo *mutations is then estimated as *2N_o _*because only half of them are expected to be transmitted. Because humans have a pair chromosomes, the mutation rate *µ *is estimated as *N_0_/L*. For a type II family, mutation rate *µ *is estimated as *2N_o_/3L *because 75% of *de novo *mutations are expected to transmit to 1 of the 2 children. As families **d **and **e **have both parents with sequencing data available, it is possible to further exclude any of variants present in both parents, further reducing the false discovery rate.

## Results

We analyzed the sequencing data after quality controls provided by GAW18. By investigating the first 2 generations in the 8 families, we were able to identify a total of 13,584 *de novo *mutation candidates. Among these candidates, 186 were successfully transmitted to the grandchildren. On average, 23.25 (±5.62) *de novo *mutations on the odd autosomes per family were discovered (Table 1). Considering there is an average of 1.35 billion base pairs on the odd chromosomes, we estimated an average mutation rate (*µ*) of 1.64(±0.42) × 10^−8 ^per position per haploid genome, which falls in the range between 1.1 × 10^−8 ^and 3.8 × 10^−8 ^reported in the literature [4-6]. We did not observe a significant difference between the *de novo *mutations in males (1.61 × 10^−8 ^) and females (1.67 × 10^−8^).

**Table 1:** Summary of *de novo *mutation numbers in each family.

Family ID	Family type	Paternal age	Maternal age	Observed *de novo *mutations *N_o_*	*De novo *mutation rate *µ*
Fam2_1	e	35	31	27	2.00 × 10^−8^
Fam2_2	a	26	24	25	1.85 × 10^−8^
Fam2_3	c	25	23	27	1.33 × 10^−8^
Fam10_1	d	29	23	33	2.44 × 10^−8^
Fam10_2	b	26	29	20	1.48 × 10^−8^
Fam10_3	b	21	25	19	1.41 × 10^−8^
Fam16_1	e	31	27	18	1.33 × 10^−8^
Fam27_1	e	26	21	17	1.26 × 10^−8^

Average					1.64(±0.42) × 10^−8^

We used the UCSC genome browser (http://genome.ucsc.edu/) [7,8] and SIFT (http://sift.jcvi.org/) [9] to map and predict the protein functions of the 186 *de novo *mutations. Seven of them are in exon regions and 2 are nonsynonymous SNVs. One of the nonsynonymous SNVs is in the gene *PDZ domain containing 2 *(*PDZD2*) on chromosome 5; the other is in gene spastic ataxia of *Charlevoix-Saguenay (sacsin) *(*SACS*) on chromosome 13. *PDZ *domains are protein-protein recognition modules that play a central role in organizing diverse cell signaling assemblies, most often in the cytoplasmic tails of transmembrane receptors and channels. *PDZD2 *and its secreted form (*sPDZD2*) are possibly involved in functional maturation of human fetal PPC-derived ICCs and the early stages of prostate tumorigenesis [10,11]. *SACS *encodes the sacsin protein, which is highly expressed in the central nervous system. Mutations in this gene will cause autosomal recessive spastic ataxia of Charlevoix-Saguenay, but the detail of its function is still unknown [12,13].

CpG sites are known as the mutation hotspots in mammals [14]. In the great apes, the *de novo *mutation rate on the CpG sites is estimated to be 11 times higher than that on the non-CpG sites [4,15]. We extracted the CpG islands from UCSC genome browser and examined the locations of the identified *de novo *mutations. Of our confirmed 186 *de novo *mutations, only 1 is located on the CpG islands. Considering the coverage of CpG islands on the odd autosomes, we expect we underestimated the CpG mutations. In the remaining 185 non-CpG mutations, we observed 127 transition mutations and 58 transversion mutations. The transition-to-transversion ratio is 2.2, similar to previous estimates [4,6].

Furthermore, we examined the relationships between the age of parents and the *de novo *mutation rate in the child using the first 2 generations in the 8 families by constructing linear models. In general, the *de novo *mutation rate in the child increases with the child's parents' ages, especially with the father's age. This is consistent with the previous report that the *de novo *mutation rate in offspring is positively correlated with the paternal age [1]. Nevertheless, no significant association effect was observed because of the small sample size in this study.

## Discussion

We conducted an analysis of the whole genome sequences on odd autosomes of 8 three-generation families to identify *de novo *mutations. We found this 3-generation approach is efficient, although no further resequencing of the candidate variants was performed. In the 8 selected Mexican American families, we estimated a mutation rate of 1.64(±0.42) × 10^−8 ^per position per haploid human genome, which is consistent with the previous estimates [4-6].

Among the 13,584 *de novo *mutation candidates observed in 8 three-generation families, only 186 are observed in grandchildren. This is remarkably less than the expected number of transmissions, suggesting that most *de novo *mutation candidates can be attributed to SNV calling errors. Because the goals in a whole genome sequencing project are to detect rare and possible *de novo *variants and test for association of these to a complex disease, how to account for the false-positive calls of SNVs is extremely important in an association study. Our analysis suggests sequencing family members is an efficient way to detect these SNV calling errors. For example, our analysis suggests that a variant observed in offspring but not in their parents in a simple trio can usually be treated as an SNV calling error, and should be excluded in downstream analyses. Previous studies suggest family data has many statistical advantages in detecting rare disease variants [16,17]. Thus, our results suggest whole-genome sequencing family members is worthwhile when most current whole genome sequencing projects only focus on unrelated subjects. It should be pointed out that the recruitment of multigeneration pedigrees is more difficult than family trios. However, many multigeneration pedigrees have already been collected in traditional linkage studies, such as the pedigrees used here. We expect the proposed method can be useful in detecting *de novo *mutations.

## Competing interests

The authors declare that they have no competing interests.

## Authors' contributions

XZ designed the overall study, HW conducted statistical analyses, HW and XZ drafted the manuscript. All authors read and approved the final manuscript.

## References

[B1] Kong A Frigge ML Masson G Besenbacher S Sulem P Magnusson G Gudjonsson SA Sigurdsson A Jonasdottir A Wong WS Rate of de novo mutations and the importance of father's age to disease risk Nature 2012 488 471 475 10.1038/nature11396 22914163 PMC3548427

[B2] Sebat J Lakshmi B Malhotra D Troge J Lese-Martin C Walsh T Yamrom B Yoon S Krasnitz A Kendall J Strong association of de novo copy number mutations with autism Science 2007 316 445 449 10.1126/science.1138659 17363630 PMC2993504

[B3] 1000 Genomes Project Consortium Abecasis GR Altshuler D Auton A Brooks LD Durbin RM Gibbs RA Hurles ME McVean GA A map of human genome variation from population-scale sequencing Nature 2010 467 1061 1073 10.1038/nature09534 20981092 PMC3042601

[B4] Roach JC Glusman G Smit AF Huff CD Hubley R Shannon PT Rowen L Pant KP Goodman N Bamshad M Analysis of genetic inheritance in a family quartet by whole-genome sequencing Science 2010 328 636 639 10.1126/science.1186802 20220176 PMC3037280

[B5] Kondrashov AS Direct estimates of human per nucleotide mutation rates at 20 loci causing mendelian diseases Hum Mutat 2003 21 12 27 10.1002/humu.10147 12497628

[B6] Nachman MW Crowell SL Estimate of the mutation rate per nucleotide in humans Genetics 2000 156 297 304 10978293 10.1093/genetics/156.1.297 PMC1461236

[B7] Fujita PA Rhead B Zweig AS Hinrichs AS Karolchik D Cline MS Goldman M Barber GP Clawson H Coelho A The UCSC Genome Browser database: update 2011 Nucleic Acids Res 2011 39 D876 D882 10.1093/nar/gkq963 20959295 PMC3242726

[B8] Karolchik D Hinrichs AS Furey TS Roskin KM Sugnet CW Haussler D Kent WJ The UCSC Table Browser data retrieval tool Nucleic Acids Res 2004 32 D493 D496 10.1093/nar/gkh103 14681465 PMC308837

[B9] Kumar P Henikoff S Ng PC Predicting the effects of coding non-synonymous variants on protein function using the SIFT algorithm Nat Protoc 2009 4 1073 1081 10.1038/nprot.2009.86 19561590

[B10] Leung KK Suen PM Lau TK Ko WH Yao KM Leung PS PDZ-domain containing-2 (PDZD2) drives the maturity of human fetal pancreatic progenitor-derived islet-like cell clusters with functional responsiveness against membrane depolarization Stem Cells Dev 2009 18 979 990 10.1089/scd.2008.0325 19046020

[B11] Harris BZ Lim WA Mechanism and role of PDZ domains in signaling complex assembly J Cell Sci 2001 114 3219 3231 11591811 10.1242/jcs.114.18.3219

[B12] Grieco GS Malandrini A Comanducci G Leuzzi V Valoppi M Tessa A Palmeri S Benedetti L Pierallini A Gambelli S Novel SACS mutations in autosomal recessive spastic ataxia of Charlevoix-Saguenay type Neurology 2004 62 103 106 10.1212/01.WNL.0000104491.66816.77 14718707

[B13] Engert JC Berube P Mercier J Dore C Lepage P Ge B Bouchard JP Mathieu J Melancon SB Schalling M ARSACS, a spastic ataxia common in northeastern Quebec, is caused by mutations in a new gene encoding an 11.5-kb ORF Nat Genet 2000 24 120 125 10.1038/72769 10655055

[B14] Coulondre C Miller JH Farabaugh PJ Gilbert W Molecular basis of base substitution hotspots in *Escherichia coli* Nature 1978 274 775 780 10.1038/274775a0 355893

[B15] Chimpanzee Sequencing and Analysis Consortium Initial sequence of the chimpanzee genome and comparison with the human genome Nature 2005 437 69 87 10.1038/nature04072 16136131

[B16] Zhu X Feng T Li Y Lu Q Elston RC Detecting rare variants for complex traits using family and unrelated data Genet Epidemiol 2010 34 171 187 10.1002/gepi.20449 19847924 PMC2811752

[B17] Feng T Elston RC Zhu X Detecting rare and common variants for complex traits: sibpair and odds ratio weighted sum statistics (SPWSS, ORWSS) Genet Epidemiol 2011 35 398 409 10.1002/gepi.20588 21594893 PMC3114642

